# ﻿Resurrection of *Cyrtandra kipahuluensis* (Gesneriaceae)

**DOI:** 10.3897/phytokeys.263.155411

**Published:** 2025-09-15

**Authors:** Destiny Brokaw, Hank Oppenheimer, Warren L. Wagner, Eric H. Roalson

**Affiliations:** 1 School of Biological Sciences, Washington State University, Pullman, WA 99164-4236, USA Washington State University Pullman United States of America; 2 Plant Extinction Prevention Program, Pacific Cooperative Studies Unit, University of Hawaii, PO Box 909, Makawao, HI 96768, USA University of Hawaii Makawao United States of America; 3 Department of Botany, Smithsonian Institution, P.O. Box 37012, Washington, DC, 20013-7012, USA Smithsonian Institution Washington United States of America

**Keywords:** *
Cyrtandra
*, Gesneriaceae, Hawaiian Islands, Maui

## Abstract

Recent fieldwork has revealed multiple populations in several localities that correspond morphologically to the type of *Cyrtandra kipahuluensis* H. St. John, collected by C.N. Forbes in 1919. Since the type was the only collection of this species until recent discoveries, and because it had an atypical zygomorphic calyx morphology, the specimen was considered to be a putative hybrid by Wagner et al. (1990, 1999). However, within the past decade, this distinctive calyx morphology has been found to be consistent, occurring in numerous populations across East Maui and in a single locality in West Maui. These new data provide substantial evidence that these populations should be recognized as a distinctive species.

## ﻿Introduction

The genus *Cyrtandra* J.R.Forst. & G.Forst. (Gesneriaceae) in the Hawaiian Islands is characterized by extensive polymorphism, often within a very limited geographical area. It is not uncommon to find two or sometimes several species intermingled or occurring in close proximity to one another. Within some valleys across the full elevational range, as many as eight species may occur together, such as in the well-collected Kipapa Gulch, O‘ahu. In virtually all of the mixed populations that have been examined closely, rare individuals with intermediate morphological features can be found, usually between species of the Hawaiian lineage. These have been interpreted as interspecific hybrids (Wagner et al. 1990). The study by Wagner et al. (1990) indicates that chance sympatric occurrences have provided numerous opportunities for interspecific hybridization. In ongoing research on the Hawaiian lineage, as many as 95 putative hybrid combinations have been detected. The assumption that a plant is a putative hybrid within Hawaiian *Cyrtandra* is based on the following criteria: (1) intermediate morphological features; (2) presence of putative parental species with the proper combination of features in the same geographical area; and (3) occasionally depressed pollen fertility. Depressed pollen fertility, however, has not always been found in putative hybrids and, in all cases studied to date, has never been more than moderately depressed. How highly sympatric species are maintained despite producing at least partially fertile hybrids remains a primary question of our ongoing research.

Harold St. John published, in a long series of works, more than 400 species of Hawaiian *Cyrtandra* using a narrow species concept. Among these, many names were based on a single or a few collections and included numerous putative hybrids according to the criteria of Wagner et al. (1990). *Cyrtandra kipahuluensis* H. St. John was published in 1971 based on a single collection made by C.N. Forbes in 1919 in Kipahulu Valley, East Maui. The description of *C. kipahuluensis* was based on this single collection and featured a unique zygomorphic calyx morphology, in which two lobes are separated nearly to the calyx base and the other three are connate for more than half their length. Since this morphology was otherwise unknown in Hawaiian *Cyrtandra*, Wagner et al. (1990, 1999) hypothesized that it likely represented a hybrid between a species group characterized by a zygomorphic calyx that is fusiform in bud and deciduous after anthesis, with lobes dissimilar in shape and unequal, and a species with the most common actinomorphic calyx morphology, in which all lobes are divided to the base or partway and are persistent in fruit. The only two species with known sympatry in Kipahulu Valley that could possibly produce such a unique calyx morphology were C. paludosa Gaud. var. paludosa and *C. spathulata* H. St. John (see Hybridization section for more details).

Recent fieldwork by Hank Oppenheimer and Ken Wood on East and West Maui has provided additional collections from several localities with the same distinctive calyx characters as the type collection of *C. kipahuluensis*. These collections make it clear that this distinct morphology is common in these valleys on Maui and therefore cannot represent a unique interspecific hybrid but rather a distinctive species.

In the same publication, St. John (1971) described a second species, *C. rotata*, from another collection in nearly the same locality. In the assessment of Wagner et al. (1990), this name was considered to represent a variation of *C. grayi* C.B. Clarke. After work was initiated to assess the morphology and distribution of *C. kipahuluensis* for this paper, we re-examined all *Cyrtandra* specimens from Kipahulu Valley. Upon more careful examination, in searching for the unique two distinct and three partially connate calyx lobes, we realized that their presence in the type of *C. rotata* was not due to tardy separation of the three calyx lobes but represented the same character as in the type of *C. kipahuluensis*. Both type collections therefore exhibit the same unique calyx morphology, with no other distinguishing feature unique to either collection. Based on the unique characters they share, along with locality, we conclude that they represent the same species with equal nomenclatural priority. We here select the name based on the locality as the accepted name, since the other is a reference to corolla lobes, which are similar in many species of the genus.

## ﻿Methods

All measurements were taken from dried herbarium specimens and are presented in the description as follows: length × width, followed by units of measurement (mm or cm). Variables such as plant height, associated species, threats, and calyx color were evaluated based on field observations by the authors and from herbarium specimen labels. *Cyrtandra* specimens were evaluated physically, with a few also examined digitally from the A, BISH, E, HALE, K, NY, PTBG, and US herbaria. The extent of occurrence (EOO) and area of occupancy (AOO) for *C. kipahuluensis* were calculated using ArcMap 10.6.1 (Esri 2018), based on coordinates recorded during herbarium specimen collection or field observations.

## ﻿Taxonomic treatment

### ﻿ Cyrtandra kipahuluensis

Taxon classificationPlantaeLamialesGesneriaceae

H.St. John, Pacific Sci. 25: 52. 1971.

1E420BF0-DDAE-5DD5-9787-B61FEBAF13BB

Figs 1, 2

Cyrtandra rotata H.St. John, Pacific Sci. 25: 56. 1971. Type. Hawaiian Islands: • Maui, East Maui: Ridge right side, Kipahulu [Valley], 17 Nov 1919, *C. N. Forbes 1662.M* (holotype: BISH 474907!).

#### Type.

Hawaiian Islands, Maui, East Maui: • Ridge on right side of Kipahulu Valley, 20 Nov 1919, *C. N. Forbes 1692.M* (holotype: BISH 77822!; isotypes: A!, BISH!, E!, K!).

**Figure 1.**
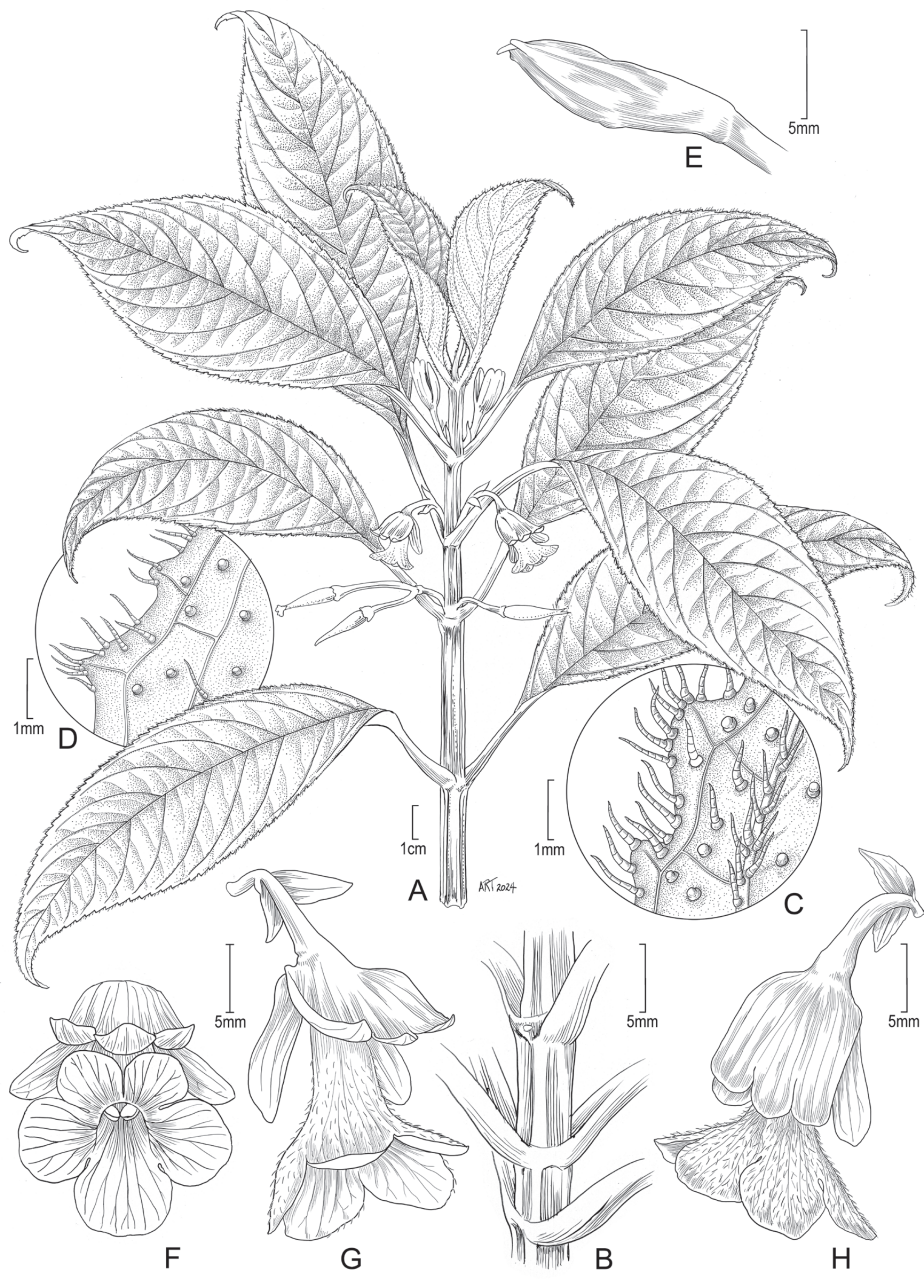
*Cyrtandra kipahuluensis* H. St. John. A. Habit showing solitary or few-flowered cymes; B. Stem section showing quadrangular stem common in the species; C. Lower leaf surface pubescence of leaf margin in East Maui populations; D. Lower leaf surface pubescence of leaf margin in West Maui populations; E. Young calyx in bud face view of flower; F. Flower, face view; G. Flower, lateral view showing the 2-lipped calyx and corolla flower; H. Flower, view from the dorsal side showing the 3 mostly connate calyx lobes. Drawn from *Oppenheimer et al. H61524* (US) from East Maui, Wailua Iki steam (A), from *Oppenheimer et al. H61518* (US) from East Maui, Kano Stream (C), from *Wood et al. 6105-A* (US) from West Maui, Waihee Valley (D), from photos by H. Oppenheimer from East Maui, Waihoi Valley, 20171227 #2 (B), 20171227 #5 (E), 20140923 #7 (F, G), 20140923 #8 (H). Illustration by Alice Tangerini.

#### Description.

Shrubs 0.75–1.75 m tall; stems square, rarely (West Maui) terete, unbranched to few-branched. Leaves opposite, ± well-spaced; those at a node slightly unequal, symmetrical, thin, chartaceous, elliptic, occasionally elliptic-ovate or elliptic-oblanceolate, 8.5–28.1 cm long, 3–11.3 cm wide, upper surface sparsely to moderately brown hirtellous, lower surface sparsely to moderately pilose to hirtellous mainly along primary and secondary veins, margins finely serrate, apex broadly acuminate, base short to long attenuate, petioles (1.1–)2.2–6.6(–9.3) cm long. Flowers solitary, occasionally 2(–5) in cymes arising in the leaf axils, occasionally along stems, sparsely to moderately brown pilose, peduncles 3–15(–21) mm long, pedicels 10–20(–24–25) mm long, bracts foliaceous, oblanceolate to narrowly elliptic, 5–12 mm long; calyx zygomorphic, persistent after anthesis, in anthesis light green to whitish green, 10–21 mm long, glabrous to occasionally moderately brown pilose primarily along veins, occasionally glandular within, upper lip consisting of 3, ¾ connate lobes 10–21 mm long; lower lip consisting of 2 lobes separating nearly to the base, 10–21 mm long; apex of all lobes rounded; corolla white, tube narrowly funnelform, curved near middle, 16–27 mm long, ca. 3–7(–9) mm in diameter medially, adaxial surface short brown pilose, upper lobes broadly elliptic, 4.5–10(–20) mm long, 5–7 mm wide, lateral lobes broadly elliptic, 4–11 mm long, 5.5–8 mm wide, lower lobes broadly elliptic, 4–11 mm long, 4–6 mm wide; ovary glabrous; style ca. 2.5–5 mm long, glabrous. Berries white, ellipsoid, ca. 2 cm long, glabrous, when dry minutely papillate at high magnification. Seeds ca. 0.05 mm long.

**Figure 2.**
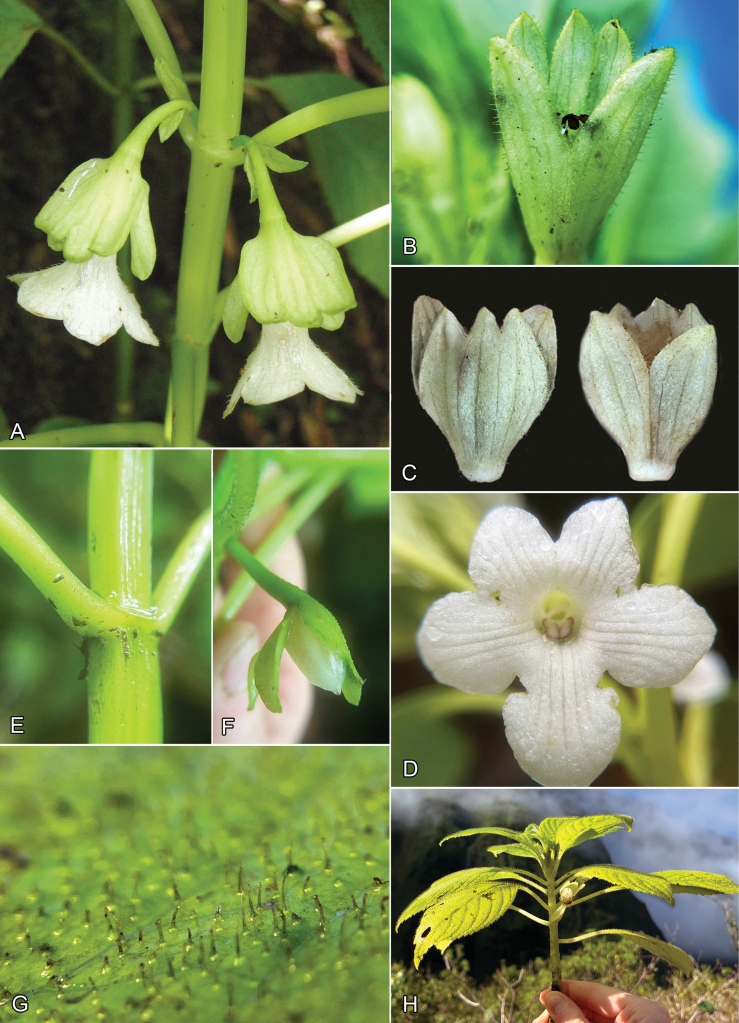
*Cyrtandra kipahuluensis* H. St. John. A. Solitary flowers with upper 3 calyx lobes connate, Waihoi Valley; B. Lower calyx lobes split to the base, Waihe’e Valley; C. Comparison of dorsal and ventral calyx lobes, Waihe’e Valley; D. Face view of corolla, Waihoi Valley; E. Square stems of East Maui specimens, Waihoi Valley, Brokaw 182; F. Persistent calyx after anthesis, Waihoi Valley, Brokaw 182; G. Pubescence on upper leaf surface, Waihe’e Valley; H. Habit, Waihe’e Valley. Photo A by Hank Oppenheimer. Photos B–H. by Destiny Brokaw.

#### Specimens examined.

**Hawaiian Islands, Maui: East Maui: Hana District**: • Kipahulu Valley, lower shelf, southwest of Fern Camp, across central pali stream, 846 m, 26 July 2017, *Oppenheimer H71706* (HALE); • Kaapahu, 23 August 1995, *Medeiros & Chimera* (HALE); • Ko’olau Forest Reserve, western tributary headwaters of Waiokamilo Stream, along small streams in damp, mossy gulch bottoms, 1071 m, 23 Oct 2007, *Oppenheimer et al. H100715* (US); • West fork of East Wailua Iki Stream, 671 m, 26 Jun 2015, *Oppenheimer & Bustamente H61524* (PTBG [2], US), 1189 m, 29 Nov 2007, *Wood & Oppenheimer 12654* (PTBG); • Wailua Nui region, headwaters above Waiokamilo and Kano Stream, 1158 m, 24 Oct 2007, *Wood et al. 12601* (PTBG), 1158 m, 24 Oct 2007, *Wood et al. 12597* (PTBG, US); • Tributary of west Wailua iki, 785 m, 19 Dec 2019, *Oppenheimer & Severson H121905* (PTBG); • Kano Stream, 671 m, 24 Jun 2015, *Oppenheimer & Bustamente H61518* (PTBG, US); • Hana Forest Reserve, Waihoi Valley, 1,064 m, 13 Mar 2024, *Brokaw et al. 182* (BISH, NY, PTBG, US); • Below ungulate control fence downstream from waterfall on south side of valley, base of pali below Kaumakani, 884 m, 23 Sep 2014, *Oppenheimer H91413* (PTBG); • South side, 27 Dec 2017, *Oppenheimer H121706* (US); • Hanawi Natural Area Reserve, Kuhiwa Valley, Kuhiwa Stream, 1044 m, 3 Dec 2013, *Oppenheimer et al. H121304* (PTBG); • Western Kuhiwa drainage basin, 1109 m, 2 Mar 2021, *Oppenheimer H32102* (PTBG); • Koolau Gap, Keanae Valley, east Piinaau drainages, 25 Sep 2007, *Wood et al. 12567* (PTBG); • Keanae Valley, east Piinaau drainages, 914 m, 25 Sep 2007, *Wood et al. 12571* (PTBG); • Kipahulu, Palikea Drainage, 890–930 m, 20 May 1994, *Wood et al. 3205* (PTBG); **West Maui: Wailuku District**: • Waihee Valley, very back of valley, 893 m, 26–27 Mar 1997, *Wood et al. 6121* (PTBG); • Very back of valley, 26–27 Mar 1997, *Wood et al. 6105-A* (US); • Waihee Valley, 776 m, 12 Mar 2024, *Brokaw et al. 161* (BISH); *Brokaw et al. 162* (US); *Brokaw et al. 163* (PTBG); *Brokaw et al. 164* (US); *Brokaw et al. 168* (US); • Back of valley on large flat plateau, wet forest, 777 m, 17 Nov 2011, *Oppenheimer et al. H111109* (PTBG); • South side, in open, muddy area formerly disturbed by feral pigs, 786 m, 16 Feb 2005, *Oppenheimer & Hansen H20506* (PTBG); • Back of valley below Kahoolewa ridge on south side of stream, 803 m, 19 May 2009, *Perlman & Oppenheimer 21640* (PTBG).

#### Phenology.

*Cyrtandra kipahuluensis* has been observed to be flowering in March, May, June, and from September to December, with fruit maturation observed in March and December.

#### Etymology.

The specific epithet is derived from the type locality, Kipahulu Valley, followed by the Latin suffix “-ensis” to indicate origin.

#### Discussion.

***Affinities*.** In the most recent full treatment of Hawaiian species of *Cyrtandra* (Wagner et al. 1990, 1999), six sections were recognized following the work of Clarke (1883), Hillebrand (1888), and St. John (1966). These were based on shared morphological characters among members of each of the sections: *Verticillatae* H. St. John, *Cylindrocalyces* Hillebr., *Crotonocalyces* Hillebr., *Apertae* C.B. Clarke, *Macrosepalae* C.B. Clarke, and *Chaetocalyces* Hillebr.

*Cyrtandra kipahuluensis* appears to be morphologically similar to species in sect. Macrosepalae C.B. Clarke due to its cylindrical bud that gradually tapers to an attenuate apex and its persistent calyx after anthesis. The only species currently classified in *Macrosepalae* C.B.Clarke in Maui are *C. grayana* Hillebr., *C. grayi*, *C. hashimotoi* Rock, and *C. spathulata*. *Cyrtandra grayana*, *C. grayi*, and *C. hashimotoi* are similar in having calyces and lobes divided to the base, as in the two lower calyx lobes of *C. kipahuluensis*, but differ in being actinomorphic or nearly so. The last species in this section on Maui, *C. spathulata*, is compared and contrasted alongside *C. macrocalyx* Hillebr. in Table 1. *Cyrtandra macrocalyx* is included in this table because the initial specimen that initiated this study was originally misidentified as *C. macrocalyx* due to similarities of the calyx in this species to that of *C. kipahuluensis*.

**Table 1.** Morphological comparisons of two similar *Cyrtandra* species to *C. kipahuluensis*.

Character	*Cyrtandra kipahuluensis*	*Cyrtandra spathulata*	*Cyrtandra macrocalyx*
Leaf dimensions (cm)	8.5–28.1 × 3–11.3	(7.5–)10–25(–41) × (2.8–) 4–7 (–13.5)	7–16 × 2.5–3.5
Leaf indumentum	Upper surface sparsely to moderately brown hirtellous, lower surface sparsely to moderately appressed pilose mainly along primary and secondary veins	Upper surface sparsely hirtellous, lower surface moderately to sparsely velvety short-pilose	Upper surface sparsely bullate-hirsute to occasionally hirtellous, lower surface moderately velvety short-pilose
Inflorescence	Flowers solitary, occasionally 2(–5) in cymes arising from leaf axils	(2–)4–7(–9) in open umbelliform cymes arising from leaf axils	Flowers solitary or rarely 2 in cymes arising in the leaf axils
Peduncle length (mm)	3–15(–21)	10–28	8–18(–25)
Pedicel length (mm)	10–20(–25)	8–19	12–34
Calyx	Zygomorphic; 10–21 mm long, divided into 3 ¾ connate upper lobes and 2 lower lobes divided nearly to the base	Nearly actinomorphic, 10–16 mm long, all lobes cleft nearly to base	Nearly actinomorphic, (17–)20–27 mm long, all lobes cleft ¼–½ their length

#### Distribution and ecology

**Figure 3.**
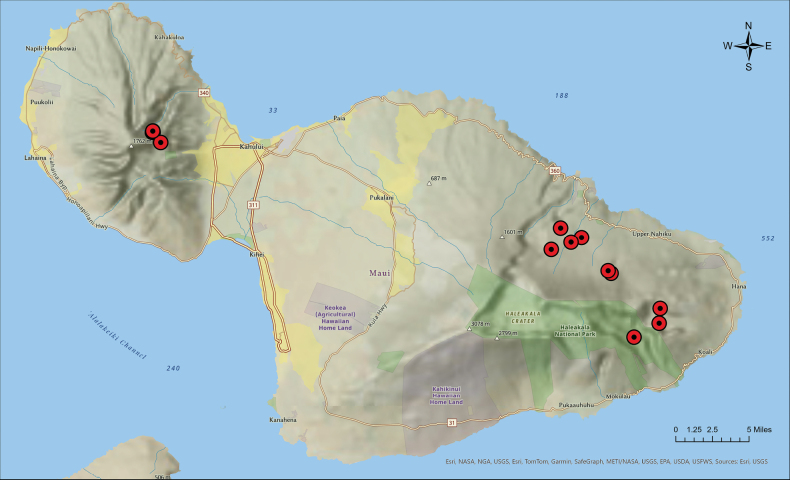
Map of *Cyrtandra kipahuluensis* occurrences indicated by red dots. Map generated via ArcGIS Pro Version 3.1 (Esri 2023).

**(Fig. 3).** Occurring in lowland and montane wet forests and gulches of East Maui from Waihoi Valley, Hanawi Natural Area Reserve, and Ko’olau Forest Reserve, and from West Maui, in Waihe’e Valley. *Cyrtandra kipahuluensis* occurs in *Metrosideros* Banks ex Gaertn. (Myrtaceae) and *Acacia koa* A. Gray montane wet forest at ca. 670–1190 m elevation. The habitat of *C. kipahuluensis* is typically dominated by Cheirodendron trigynum (Gaudich.) A. Heller subsp. trigynum, species of *Geniostoma* (Loganiaceae), *Diplazium* Sw. (Athyriaceae), Clermontia arborescens (H. Mann) Hillebr. subsp. waihiae (Wawra) Lammers, *Clermontia micrantha* (Hillebr.) Rock (Campanulaceae), *Perrottetia sandwicensis* A. Gray (Dipentodontaceae), *Hydrangea arguta* (Gaudich.) Y. De Smet & Granados (Hydrangeaceae), *Peperomia* Ruiz & Pav. (Piperaceae), *Dicranopteris* Bernh. (Polypodiaceae), *Coprosma ochracea* W.R.B. Oliv., *Kadua* Cham. & Schltdl., *Psychotria* L. (Rubiaceae), *Melicope clusiifolia* (A. Gray) T.G. Hartley & B.C. Stone, *Melicope molokaiensis* (Hillebr.) T.G. Hartley & B.C. Stone, *Melicope ovalis* (H. St. John) T.G. Hartley & B.C. Stone (Rutaceae), *Pipturus albidus* (Hook. & Arn.) A. Gray (Urticaceae), and *Machaerina gahniiformis* Gaudich.

##### ﻿Hybridization

Wagner et al. (1990) previously considered the type to be a putative hybrid between *C. spathulata* and C. paludosa var. paludosa. In the case of the type of *C. kipahuluensis*, it was the only known collection at the time with the unique calyx morphology and thought to be a hybrid between a species with an actinomorphic calyx, *C. spathulata*, and one with a zygomorphic calyx, C. paludosa var. paludosa. With the discovery of other localities of plants with this unique calyx morphology and numerous individuals with consistent characters in populations, it is evident that the type of *C. kipahuluensis* is not a putative hybrid. Further exploration has led to a number of known populations of *C. kipahuluensis* that occur sympatrically with a number of other *Cyrtandra* species (*C. grayana* Hillebr., *C. grayi*, *C. hashimotoi* Rock, *C. hawaiensis* C.B.Clarke, C. paludosa var. paludosa, *C. platyphylla* A.Gray, and *C. spathulata*).

Potential hybrids with *C. kipahuluensis* have limited observations, with variation in the number of flowers per cyme that may be a result of hybridization with species in its vicinity but may also simply represent morphological variation within the species. Putative hybridization has been hypothesized with *C. hashimotoi*, C. paludosa var. paludosa, *C. platyphylla*, and *C. spathulata* based on collections with intermediate morphology growing sympatrically with the putative parents.

##### ﻿Putative hybrid specimens examined

The designations of most hybrids are hypotheses in need of further testing. There is not a current understanding of the direction of crosses; thus, the putative hybrids here are listed in an alphabetic manner.

###### ﻿*C. hashimotoi* × *C. kipahuluensis*

**Specimen examined.** West Maui, Wailuku District, Waihe’e Valley, 784 m, *Brokaw 169* (US).

###### ﻿*C. kipahuluensis* × C. paludosa var. paludosa

**Specimen examined.** West Maui, Wailuku District, Waihe’e Valley, 810 m, *Brokaw 174* (BISH), *Brokaw 176* (BISH).

###### ﻿*C. kipahuluensis* × *C. platyphylla*

**Specimen examined.** West Maui, Wailuku District, Waihe’e Valley, 810 m, *Brokaw 175* (BISH).

###### ﻿*C. kipahuluensis* × *C. spathulata*

**Specimen examined.** East Maui, Hana District, Waihoi Valley, 1,124 m, *Brokaw 179* (BISH).

## ﻿Conservation

### ﻿Preliminary conservation assessment

*Cyrtandra kipahuluensis* falls into the Endangered (EN) category according to the IUCN criteria (IUCN 2024), EN (B1ab(iii)+2ab(iii), which reflects an EOO of 250 km^2^ and AOO of 16 km^2^, only four small disjunct populations across East and West Maui (the furthest separated by over 40 km), and a total of no more than 1000 mature plants. The continued decline in quality of habitat for *C. kipahuluensis* is evidenced by severe habitat degradation from invasive non-native mammals such as pigs (*Sus scrofa* L.) and rats (*Rattus* spp.), along with introduced slugs, insects, and diseases. Specific invasive, non-native plants observed in these habitats include *Ageratina adenophora* Spreng. (Asteraceae), *Sphaeropteris cooperi* (Hook. ex F.Muell.) R.M.Tryon (Cyatheaceae), *Angiopteris evecta* (C.Presl) Baker (Marattiaceae), *Chaetogastra herbacea* (DC.) P.J.F.Guim. & Michelang., *Miconia crenata* (Vahl.) Michelang. (both Melastomataceae), *Psidium cattleyanum* Sabine (Myrtaceae), *Axonopus fissifolius* (Raddi) Kuhlm., *Cortaderia jubata* (Lemoine) Stapf, *Paspalum urvillei* Steud. (all Poaceae), *Buddleja asiatica* Lour. (Scrophulariaceae), and *Hedychium gardnerianum* Sheppard ex Ker Gawl. (Zingiberaceae).

### ﻿Conservation efforts

There are no specific activities to protect *C. kipahuluensis*. However, Haleakalā National Park, the State of Hawai’i, and both the East Maui Watershed Partnership and Mauna Kahalawai (aka West Maui) Watershed Partnerships have constructed ungulate exclusion fences to protect habitat from damage by feral pigs (*Sus scrofa*). More recently some of these fences have been retrofitted to exclude an expanding range of axis deer (*Axis axis* Erxleben). These fences are on varying schedules of inspection and, if necessary, repair. However, these efforts have not been fully successful, nor do they include all currently occupied habitat. A continued decline in habitat quality, mainly conversion by non-native vascular plant species and proliferation of non-native slugs, is expected based on personal observation (Hank Oppenheimer).

A few habitat-modifying weeds are controlled, notably *Miconia calvescens* (Melastomataceae) by the Maui Invasive Species Committee. Also controlled by MISC is Andean pampas grass (*Cortaderia jubata*, Poaceae). Some benefit is derived from other small-scale and localized control of other weeds by the National Park Service, the State of Hawaii Division of Forestry & Wildlife, Watershed Partnerships, and the Plant Extinction Prevention Program.

There are no seeds of *C. kipahuluensis* in storage at the Lyon Arboretum Seed Conservation Laboratory in Honolulu (Nate Kingsley, pers. comm., May 2025).

## Supplementary Material

XML Treatment for Cyrtandra kipahuluensis

## ﻿Additional information

### Use of AI

No use of AI was reported.

### Author ORCIDs

Hank Oppenheimer https://orcid.org/0000-0002-9416-1889

Warren L. Wagner https://orcid.org/0000-0001-5012-8422

Eric H. Roalson https://orcid.org/0000-0003-1655-3681

### Data availability

All of the data that support the findings of this study are available in the main text.

## References

[B1] Clarke CB (1883) Cyrtandreae (Gesneracearum tribus). Monographiae Phanerogamarum 5: 1–303.

[B2] Esri (2018) ArcGIS Desktop: Release 10.6.1. Environmental Systems Research Institute, Redlands, California.

[B3] Esri (2023) ArcGIS Pro (Version 3.1). Environmental Systems Research Institute, Redlands, California. https://www.esri.com/en-us/arcgis/products/arcgis-pro

[B4] Hillebrand W (1888) Flora of the Hawaiian Islands: A description of their phanerogams and vascular cryptogams. Lubrecht & Cramer, Monticello, New York, 673 pp. [Facsimile edition 1981]

[B5] IUCN (2024) Guidelines for Using the IUCN Red List Categories and Criteria. Version 16. Prepared by the Standards and Petitions Committee. https://www.iucnredlist.org/documents/RedListGuidelines.pdf

[B6] St John H (1966) Monograph of *Cyrtandra* (Gesneriaceae) on Oahu, Hawaiian Islands. Bernice P. Bishop Museum Bulletin 229: 1–465.

[B7] St John H (1971) Endemic plants of Kipahulu Valley, Maui, Hawaiian Islands. Hawaiian Plant Studies 36. Pacific Science 25: 39–79.

[B8] Wagner WL Herbst DR Sohmer SH (1990) *Cyrtandra*. Manual of the Flowering Plants of Hawai‘i (Vol. 1). University of Hawaii Press and Bishop Museum Press, Honolulu, 1853 pp. [Bishop Museum Special Publication 83]

[B9] Wagner WL Herbst DR Sohmer SH (1999) *Cyrtandra*. Manual of the Flowering Plants of Hawa i‘i (Vol. 1). Revised edition with supplement by Wagner WL, Herbst DR. University of Hawaii Press, Honolulu, 1855–1918. [Bishop Museum Special Publication 97]

